# Optimization of the Methods to Develop Stable Polymer Gels for Water Management in Medium- and Ultra-High-Salinity Reservoirs

**DOI:** 10.3390/gels9070540

**Published:** 2023-07-03

**Authors:** Shuiqing Hu, Mingchen Ding, Yafei Hu, Yefei Wang, Jiangyang Dong

**Affiliations:** 1 PetroChina Research Institute of Petroleum Exploration & Development, Beijing 100083, China; 2 School of Petroleum Engineering, China University of Petroleum (East China), Qingdao 266580, China; wangyf@upc.edu.cn (Y.W.);; 3 Key Laboratory of Unconventional Oil & Gas Development, China University of Petroleum (East China), Ministry of Education, Qingdao 266580, China; 4 Shandong Key Laboratory of Oilfield Chemistry, China University of Petroleum (East China), Qingdao 266580, China

**Keywords:** high temperature, high salinity, polymer gel, stability enhancement, polymer type, deoxidizer, complexant

## Abstract

Polymer gels suffer from a serious syneresis issue when exposed to high-temperature and high-salinity (HTHS) conditions, which limits their use as water-treatment agents in this type of reservoir. In this paper, the effects of the polymer type/concentration, deoxidizers, and stabilizers on the long-term stability of polymer gels were systematically studied; thus, the methods to develop stable polymer gels for two typical levels of salinity were optimized. The results show the following: (1) For a medium-salinity condition (TDS: 33,645.0 mg/L) at 125 °C, conventional HPAM gels completely dehydrate within only 1 day, and the addition of a deoxidizer hardly improved their stability. Some special polymers, e.g., AP-P5, MKY, and CPAM, are able to form stable gels if a high concentration of 0.8% is used; the syneresis rate of these gels is about 10% after 30 days. However, the addition of the complexant sodium oxalate significantly improves the stability of gels formed by all five of these different polymers, which behave with a 0% syneresis rate after 30 days pass. Complexants are the most economical and feasible agents to develop stable gels in medium-salinity water. (2) Gels enhanced using the methods above all become unstable in a more challenging ultra-high-saline condition (TDS: 225,068.0 mg/L). In this case, special calcium- and magnesium-resistant polymers are required to prepare stable gels, which show 0% syneresis rates after 30 days, have relatively low strengths, but do produce a good plugging effect in high-permeability cores.

## 1. Introduction

**Figure 1:**
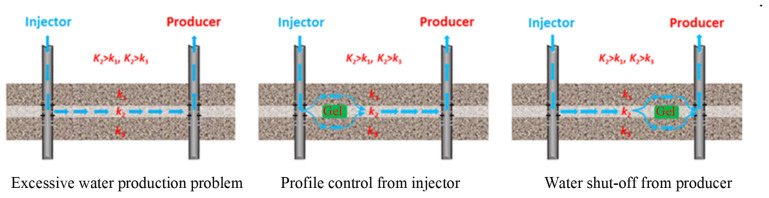
Schematic of the excessive water production problem and water management mechanisms using polymer gel [3].

Secondary oil recovery processes are often employed to extract as much oil as possible from oil wells. A common approach involves water injection. However, when this method is employed, channeling often occurs due to viscous fingering and reservoir heterogeneity. This results in great water production and a high water-cut situation in the oil well, which damages oil production [1,2]. In this case, profile-modification and water-plugging techniques are usually needed to inhibit water channeling. One solution involves using polymer gels, as shown in Figure 1 [3]. In production wells where excessive water is produced, a polymer gel can be injected to plug offending zones or areas. Meanwhile, injection well treatments via polymer gel are applied to direct the flow of the injected fluid to the oil-containing rock that was bypassed in previous water-flooding operations; thus, additional oils can be recovered. Great success has been achieved in their applications [3,4,5]. However, under high-temperature and high-salinity (HTHS) conditions, these polymer gels are easily dehydrated, so their long-term stability is poor. This severely limits their application in HTHS reservoirs.

Various methods have been explored to enhance the temperature and salt resistance of polymer gels, based mainly on the following ideas:

(1) The use of temperature and salt-resistant polymers. Two approaches are usually employed here. The first involves using a hydrophobic association polymer (e.g., AP-P4, AP-P5, MKY, etc.) or cationic-type polymer. The former type maintains a high viscosity via the association that occurs between its hydrophobic groups under HTHS conditions (which subsequently improves the stability of its corresponding gels). Using associated polymers such as AP-P4, researchers have managed to develop temperature- and salt-resistant gels that are stable at 95 °C and 16.0 × 10^4^ mg/L [6,7,8,9]. In contrast, cationic polymers are positively charged and therefore tend to exclude other cations in water (especially Ca^2+^/Mg^2+^ ions). This reduces the effect of those cations on the stability of the gel. Zhou and Xu, for example, used cationic polyacrylamide (CPAM) in place of conventional hydrolyzed polyacrylamide (HPAM) and thus developed a cationic polymer gel [10,11]. The gel was found to have good stability under the test conditions used: a temperature of 85 °C and an ultra-high salinity of 22.0 × 10^4^ mg/L (Ca^2+^/Mg^2+^: 10,000 mg/L). The second approach is to use special copolymers and terpolymers. In particular, some special temperature- and salt-resistant monomers can be introduced. In this way, for example, 2-acrylamido-2-methylpropane sulfonic acid (AMPS), *N*-vinyl pyrrolidone (NVP), *N*-vinylacetamide (NVA), dimethylaminoethyl methacrylate (DMAM), etc., can be introduced into polymers such as polyacrylamide (PAM). This inhibits the hydrolysis of PAM at high temperatures, which reduces the formation of carboxylic acid groups in the PAM polymer and significantly enhances its temperature and salt resistance. Gailard, Zhu, and Ye found that PAMs containing NVP monomers can be rendered stable even at a temperature of 150 °C [12,13,14]. The polymers remained stable for five months and hence exhibited good temperature resistance. Copolymer gels based on acrylamide and AMPS have also been focused upon by many researchers, and a whole class of polymer gels that are stable up to a maximum temperature and salinity of 130 °C and 22 × 10^4^ mg/L, respectively, have been developed [15,16,17,18,19,20,21,22]. Hsieh et al. reported that terpolymers made of AM/AMPS/NVP possessed excellent thermal stability and aged well in seawater at temperatures up to 149 °C [23]. Lu et al. prepared a polymer gel by copolymerizing acrylamide and acrylonitrile (AN) and found that it remained stable for 12 months at 105 °C [24]. The polyvinyl alcohol (PVA), phenol, and formaldehyde gel system prepared by Hoskin et al. was found to dehydrate less than 5% after aging for 70 days at 204.4 °C [25]. PVA gels have also been shown to be thermally stable by Victorius and Shu [26,27].
(2) The use of temperature and salt-resistant crosslinking agents. Gels can be prepared by replacing the traditional metal ions or phenolic system with crosslinking agents such as polyethylenimine (PEI). In this way, people have prepared polymer gels that are stable at the highest evaluation temperature used of 177 °C [28,29,30,31,32,33]. Although these gels have good temperature and salt resistance, they have an obvious deficiency: a high concentration of PEI crosslinker needs to be used, which makes this approach uneconomical.
(3) Adding a complexant to act as a stabilizer. Agents can be employed that form complexes with divalent ions and thus deactivate them. Such complexants thus resist the occurrence of gel syneresis that results from the over-crosslinking of sites featuring carboxyl functional groups caused by divalent ions. Albonico et al. systematically investigated the effect on the stability of polymers and their corresponding gels resulting from the use of more than ten complexants (including EDTA, oxalate, citrate, and NTA) in water at 120 °C and containing 1.0 × 10^4^ mg/L of salt [34]. They found that the addition of such agents significantly enhances the stability of the gel. Jia et al. and Wei et al. prepared polymer gels that were stable in seawater at 130 °C that contained 3.5 × 10^4^ mg/L of salt [35,36]. In their work, however, organophosphates and a polyate (called WZ) were used as stabilizers.

As mentioned above, the stability of a polymer gel under HTHS conditions is a complex issue dependent on numerous factors, e.g., temperature, salinity/ion type, polymer type/concentration, crosslinker type, addition of stabilizers, and so on. Various methods have thus been developed to enhance the stability of gels (by using temperature- and salt-resistant polymers, using specially crosslinkers, adding complexant as a stabilizer, and so on). However, there is still a very critical problem: it is still unclear over what temperature and salinity ranges the various methods are able to enhance the stability of a given polymer gel. Alternatively, for a specific temperature and salinity condition, it is difficult to determine which methods are most optimal and economical in achieving a stable gel (and which methods are not). In other words, what is the best and cost-optimal way to enhance the stability of a polymer gel in particular circumstances (since many of the explored methods above are too expensive to use)?

In light of these problems, the effects of temperature, salinity/ion type, polymer type/concentration, deoxidizer, and stabilizer on the long-term stability of phenolic crosslinked polymer gels were systematically studied in this work. The mechanism responsible for the syneresis of the gels in HTHS conditions could thus be clarified firstly. More importantly, the most suitable and economical approach to develop stable polymer gels in reservoirs with medium- and ultra-high salinity could be found from the evaluated four typical methods (improving polymer type/concentration, adding deoxidizer, adding stabilizer, and choosing a special calcium–magnesium-resistant polymer).

## 2. Results and Discussion

### 2.1. Syneresis Mechanism of Gel at HTHS

HTHS conditions comprise the main reason that gels experience syneresis and destruction. So, the effects of temperature and ion type/concentration on gel stability were firstly investigated. A typical phenolic crosslinked polymer gel (0.8% HPAM + 0.3% urotropine + 0.3% resorcinol) was first prepared in saline solutions with different types/concentrations of ions. The gels were maintained at temperatures in the range 60–125 °C for different periods of time, and their syneresis rates and elastic moduli were evaluated. The results obtained are shown in Table 1.

The effect of temperature was investigated first using a solution with medium salinity (TDS: 3.4 × 10^4^ mg/L, Ca^2+^/Mg^2+^: 1650 mg/L). At relatively low temperatures of 60 and 80 °C, the gel remained stable for at least 30 days, during which no syneresis was observed. The elastic moduli measured at these temperatures were 1986.0 and 1360.0 mPa, respectively. This indicates that high salt or high calcium and magnesium content, each by itself, does not necessarily lead to the syneresis and destruction of the gel at medium–low temperature. However, when the temperature was increased to 100 and 125 °C, the gels were hardly stable at all, and severe syneresis occurred only 1 day after preparation (syneresis rate of ~100%). This confirms that the commonly used phenolic crosslinked HAPM gels are difficult to stabilize for long periods of time under HTHS conditions.

The effects of ion type and concentration were subsequently explored further. Table 1 shows that when the Na^+^ concentration was varied in the range 5000–80,000 mg/L, the gel strength decreased significantly after aging for 30 days at 125 °C (the elastic modulus decreased from 6100 to 2120 mPa as the Na^+^ concentration increased). However, despite their weakened state, it is very interesting to note that the gels were not significantly dehydrated and remained stable after 30 days even when subjected to high temperature (125 °C) and high salinity (Na^+^ concentration of 80,000 mg/L). The stability of the gel varied more dramatically when the concentration of the Ca^2+^/Mg^2+^ ions was varied. Specifically, when the Ca^2+^/Mg^2+^ concentration was very low (200 and 400 mg/L), the gel was only slightly dehydrated after aging for 30 days at 125 °C. However, when the concentration was increased to 800–1600 mg/L, the gel was completely dehydrated after just 1 day. Thus, these divalent cations have a very significant effect on the stability of the polymer gel. Comparing these results, it is easy to find that the destruction of the gel under HTHS conditions is predominantly caused by both the high temperature and high Ca^2+^/Mg^2+^ content of the water. Without the divalent cations, the monovalent Na^+^ cation by itself does not lead to gel syneresis.

**Figure 2:**
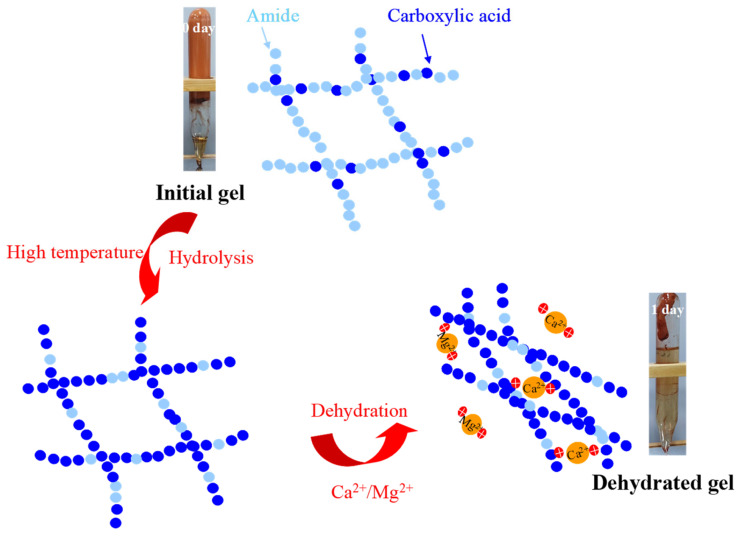
Schematic diagram illustrating the mechanism associated with gel syneresis and the statuses of the initial and dehydrated gels (with the red arrows representing the evolution direction of the gel).

The above results indicate that the key to improving the long-term stability of polymer gels under HTHS conditions is to enhance their temperature and Ca^2+^/Mg^2+^ resistance. The Ca^2+^/Mg^2+^ ions mainly affect the stability of the gel because they attract the carboxylic acid groups (formed by the hydrolysis of the polymer) under electrostatic interaction. This interaction weakens the electrostatic repulsion among the carboxylic acid groups, and as a result, molecules of the polymer curl up and the gel dehydrates. In addition, the electric neutralization between Ca^2+^/Mg^2+^ ions and carboxylic acid groups also reduces the attraction of the latter to water molecules, leading to a reduction in the water-holding capacity of the polymer. At last, gel contraction and syneresis happen, as shown in Figure 2. At high temperature, the HPAM is further hydrolyzed to form a large number of carboxylic acid groups which have significant electrostatic attraction with the divalent cations and result in the expulsion of water.

**Figure 3:**
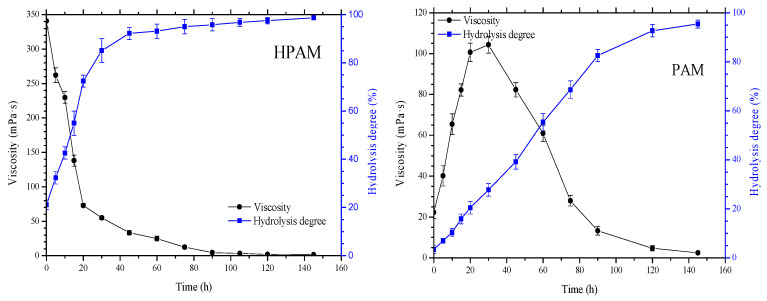
Variation of the viscosity and degree of hydrolysis of HAPM and PAM as they age.

To verify the hydrolysis state of the polymer at the experimental temperature of 125 °C, two polymers, HPAM and PAM, were hydrolyzed according to a method described in the literature [37]. As shown in Figure 3, HPAM hydrolyzes rapidly at 125 °C, and its degree of hydrolysis increases sharply from 21.3% to 91.6% after 40 h. At the same time, its viscosity decreases significantly. PAM hydrolyzes slowly, but its hydrolysis also reaches a high level of 84.0% after 100 h. This further confirms that the polymer is indeed rapidly hydrolyzed at high temperature of 125 °C.

The above results, taken together, suggest that some of the practices currently used in temperature- and salt-resistant gel research perhaps require improvement, such as the following: (1) When investigating the performance of gels in HTHS environments, some chose a single NaCl solution to prepare the polymer gel [7,38,39], while the key to affecting the application of a polymer gel in an HTHS reservoir is the gel’s stability, which is mainly controlled by temperature and Ca^2+^/Mg^2+^ content in water, rather than the Na^+^ concentration. Therefore, the effect of Ca^2+^/Mg^2+^ should be more emphasized instead of Na^+^ concentration. (2) The effect of divalent cations on the performance of polymer gels is often reported. However, such reports tend to consider only their effects on gelation time and initial colloid strength [40], while the most critical consideration—the long-term stability of the gel—is often omitted. Therefore, gel stability should be given more attention when developing temperature- and salt-resistant polymer gels (and not just gelation time and gel strength).

### 2.2. Gel Optimization for Medium-Salinity Conditions

#### 2.2.1. Effect of Polymer Type and Concentration

In light of the poor stability of the polymer gel under high-temperature and high-Ca^2+^/Mg^2+^ conditions, some typical temperature- and salt-resistant polymers (such as hydrophobic association polymer AP-P5, MKY, nonionic polymer PAM, cationic polymers CHPAM, etc.) were first chosen to improve gel stability. They were used at concentrations of 0.4% and 0.8%. A crosslinker was also used composed of 0.3% urotropine and 0.3% resorcinol. The performance of the gels was first examined under medium-saline conditions (TDS: 3.4 × 10^4^ mg/L, Ca^2+^/Mg^2+^: 1650 mg/L) at 125 °C, giving the results presented in Table 2.

Table 2 shows that the HPAM and PAM gels are all completely dehydrated after aging for 1 day. Moreover, the stability of the gels could not be enhanced by increasing the polymer concentration from 0.4% to 0.8%. Using PAM, which has a lower initial hydrolysis state compared to HPAM, also failed to significantly improve the stability of the resulting polymer gel.

The gels produced using CPAM, AP-P5, and MKY at a concentration of 0.4% were also completely dehydrated within 1–2 days, and there was no significant improvement in their stability, either. However, this was not the case when their concentrations were increased to 0.8%. As can be seen from the table, the gels produced using 0.8% polymer were only dehydrated by 10% after aging for 30 days. We further extended the evaluation time to 120 days, where those three gels remained stable, with incomplete syneresis. Thus, provided a high concentration (0.8%) of CPAM, AP-P5, or MKY was used, stable gels that are suitable for use in high-temperature (125 °C) and medium-salinity (TDS: 3.4 × 10^4^ mg/L, Ca^2+^/Mg^2+^: 1650 mg/L) environments were obtained. Moreover, among these gels, the ones produced using CPAM had the highest elastic moduli.

There are two main reasons why the gels produced using CPAM, AP-P5, and MKY are stronger and more stable than those derived from HPAM and PAM: (1) CPAM contains cationic carbamyl groups, which will electrostatically repel Ca^2+^/Mg^2+^ ions in water. This improves their poor compatibility with the carboxylic acid groups in the polymer. (We also carried out a stability study of gels produced using CPAM polymers with different degrees of cationic character: 5–80%. It was found that the CPAM gel produced with a cationic degree of 10% had the best stability, with only a small amount of syneresis occurring after aging for 30 days. If the cationic degree is too large, exceeding 20%, say, then the CPAM gel is also unable to remain stable for a long time under high-temperature and medium-salinity conditions.) (2) The hydrophobic association polymers AP-P5 and MKY both contain hydrophobic groups and a certain amount of temperature- and salt-resistant AMPS. This can enhance the structural strength of the crosslinked polymer network through association. This also slows down the rate of hydrolysis of the polymers and improves their temperature and salt resistance. It should be noted, however, that the gels formed by these three types of polymers require the use of high polymer concentrations. This, in itself, causes underlying problems such as uneconomically high costs and poor reservoir injectivity.

Focusing on CPAM, AP-P5, and MKY gels, the salinity was further increased to an ultra-high level (TDS: 22.5 × 10^4^ mg/L, Ca^2+^/Mg^2+^: 10,872 mg/L) to determine their adaptability. Unfortunately, the gels produced were completely dehydrated after aging for just 1 day even though high polymer concentrations (0.8%) were used to prepare them. Therefore, we need to look for other ways to obtain stable gels in such high-temperature and high-salinity environments instead of using a high concentration of hydrophobic association polymers or cationic polymers.

#### 2.2.2. Effect of Deoxidizer

Oxygen in water can easily lead to the rupture of molecular bonds and hence degradation of polymers at high temperature. This is detrimental to the stability of polymer gels and is considered to be one of the main reasons that gel syneresis occurs under HTHS conditions [41,42].

**Figure 4:**
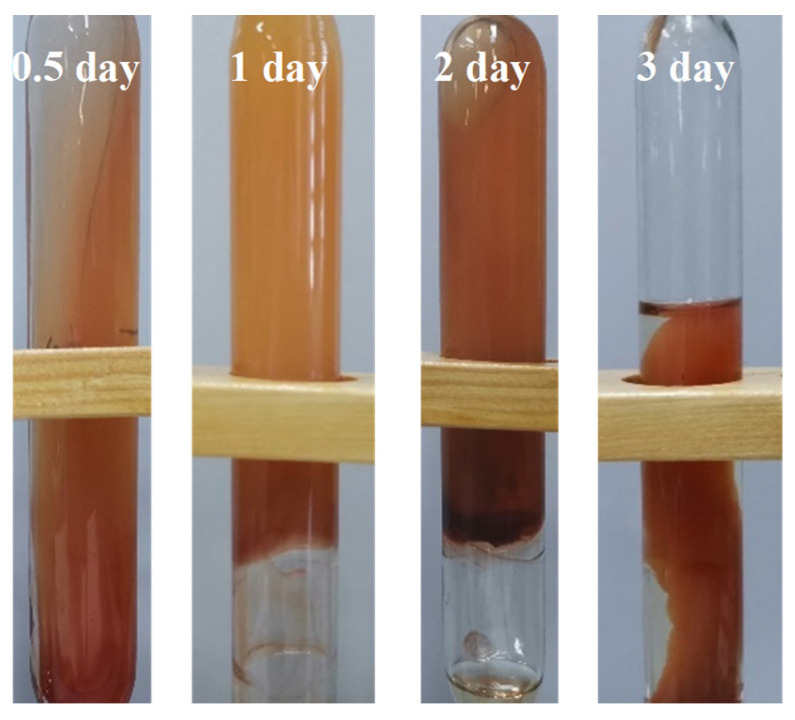
Change in morphology of a PAM gel containing thiourea over 3 days.

The deoxygenant thiourea is one of the most commonly used additives in high-temperature polymer systems. It can remove the oxygen in a solution and will hence inhibit the oxidative degradation of polyacrylamide. Therefore, the feasibility of adding a deoxygenant to the polymer gel to enhance its stability was explored. Figure 4 shows the morphology of a PAM-based gel in a solution with medium salinity observed over a period of time. The specific composition of the gel is 0.8% PAM + 0.3% urotropine + 0.3% resorcinol + 0.4% thiourea.

The addition of thiourea does indeed improve the stability of the gel. The syneresis rate after aging the gel for two days at 125 °C was found to be just 10%. However, the equivalent gel without thiourea was rapidly dehydrated and destroyed just 1 day after preparation (see Table 1 and Table 2 and Figure 2). However, by the time the gel had aged for 3 days, the syneresis rate had increased significantly (to over 50%). It can thus be concluded that the addition of a deoxygenant can indeed improve the stability of phenolic crosslinked polymer gels, to some extent. However, it cannot solve the fundamental problem of gel syneresis under HTHS conditions. The PAM gel with thiourea was significantly dehydrated after only 3 days, and so deoxygenation is not the key to improving the stability of phenolic crosslinked polymer gels.

#### 2.2.3. Effect of Complexant as a Stabilizer

The poor compatibility of carboxylate groups (formed by the hydrolysis of the amide groups in the polymer at high temperature) with Ca^2+^/Mg^2+^ ions is the root cause of gel syneresis. Therefore, adding a complexant to reduce the interaction between these divalent cations and the carboxylic acid groups should inhibit gel syneresis. This possibility was investigated by using different complexants to stabilize polymer gels subjected to a high temperature (125 °C) and both medium- and ultra-high-salinity conditions.

(1) Gels stabilized by different complexants.

Five different complexants (DTPMP, glycine, EDTA, sodium salicylate, and sodium oxalate) were used in an attempt to enhance the performance of a PAM gel system (0.8% PAM + 0.3% urotropine + 0.3% resorcinol). The concentration of the complexant added was fixed at 0.3% and the gels were evaluated at 125 °C under medium-saline conditions, giving the results shown in Table 3.

Table 3 shows that the addition of 0.3% DTPMP only enhanced the stability of the PAM polymer gel by a very limited amount, the syneresis rate reaching 90% after 30 days of aging. In contrast, without DTPMP, syneresis was complete after just 1 day (Table 2). The other four complexants, however, were able to significantly enhance the stability of the polymer gel. Among these, the best performance enhancement was achieved using sodium oxalate, which reduced the syneresis rate of the gel to just 10% after high-temperature aging for 30 days.

(2) Ability of sodium oxalate to stabilize different polymer gels.

As sodium oxalate was found to significantly enhance the stability of the PAM gel in Table 3, further experiments were performed to see if it could also stabilize other polymer gels. Thus, 0.4% sodium oxalate was used to enhance the polymer gels produced using HPAM, PAM, CPAM, AP-P5, and MKY (considering that sodium oxalate is relatively cheap, we increased its consumption to 0.4% in these trials to further improve the stability of the gels). The syneresis rates and elastic moduli thus measured are presented in Table 4.

Table 4 shows that all the polymer gels exhibit significantly enhanced stability after the addition of sodium oxalate. For a lower polymer concentration of 0.4%, HPAM, PAM, CPAM, AP-P5, and MKY gels without sodium oxalate were all completely dehydrated only 1–2 day after their preparation (Table 2), but behaved with no syneresis after 30 days in the presence of sodium oxalate (Table 4). When the polymer dosage was increased to 0.8%: (1) The HPAM and PAM gels alone still could not stabilize and were completely dehydrated in 1 day (Table 2). With sodium oxalate, however, no syneresis occurred after 30 days had passed (Table 4). (2) The CPAM, AP-P5, and MKY polymer gels made using 0.8% polymer were more stable without complexant (Table 2), their syneresis rates reaching about 10% after 30 days. With sodium oxalate, however, these rates dropped to almost 0.0%. The elastic moduli of the gels were also increased with the addition of sodium oxalate, demonstrating that their strengths were also enhanced.

Generally speaking, comparing the stability results for the five polymer gels with and without sodium oxalate, it can be seen that in a high-temperature (125 °C), medium-saline (TDS: 3.4 × 10^4^ mg/L, Ca^2+^/Mg^2+^: 1650 mg/L) environment, stable gels can be successfully obtained by adding a complexant to the gel solution. Moreover, this method worked for all five of the different types of polymer gels studied. So, we now have two methods that can be used to develop polymer gels that are stable under this medium-salinity condition, namely, using a high concentration (e.g., 0.8%) of CPAM, AP-P5, or MKY to make the gel or by adding some complexant to the gel solution. As complexants are usually relatively inexpensive (compared with polymers), the latter method may be the most economically feasible approach.

(3) Salinity adaptability of complexant-stabilized gel.

Given the excellent performance of complexants in stabilizing polymer gels in solutions with medium salinity, complexants were applied to solutions with ultra-high salinity. This is important, as stable gels are urgently needed to carry out efficient water-plugging operations in reservoirs with such conditions, e.g., the domestic Tazhong 402 and Tahe oilfields and the Ahadib carbonate reservoir in the Middle East.

**Figure 5:**
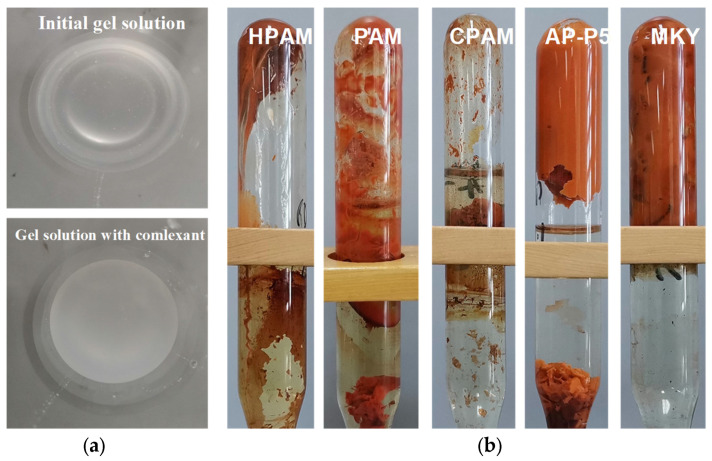
State of different gels (**a**) before and (**b**) after their gelation (for 0.5 day).

To investigate the adaptability of complexant-stabilized polymer gels to ultra-high-saline conditions (TDS: 22.5 × 10^4^ mg/L, Ca^2+^/Mg^2+^: 10,872 mg/L), five different types of polymer gels were prepared and maintained at 125 °C for stability evaluation. In each case, 0.8% polymer (HPAM, PAM, CPAM, AP-P4, and MKY) was used in combination with 0.3% urotropine, 0.3% resorcinol, and 0.4% sodium oxalate (Figure 5).

When the sodium oxalate was added to the polymer gels, the initially transparent solutions became turbid and a significant amount of precipitation occurred (Figure 5a). After gelation, it was found that all of the gels (based on HPAM, PAM, CPAM, AP-P4, and MKY) became completely dehydrated after high-temperature aging for just 0.5 day. This is very disappointing and clearly shows that the sodium oxalate was unable to stabilize the polymer gels in such ultra-high-saline conditions.

In summary, our results indicate that complexant-stabilized gels should only be used in medium-saline conditions (TDS: 3.4 × 10^4^ mg/L, Ca^2+^/Mg^2+^: 1650 mg/L) as they are unlikely to be effective when the salinity reaches ultra-high levels (TDS: 22.5 × 10^4^ mg/L, Ca^2+^/Mg^2+^: 10,872 mg/L). These results also suggest that we need to find other ways to stabilize gels intended for use in such harsh conditions.

### 2.3. Gel Optimization for Ultra-High-Salinity Conditions

As we have seen, traditional temperature- and salt-resistant polymers (e.g., CPAM, AP-P5, and MKY) are not suitable for use in harsh conditions (125 °C; TDS: 22.5 × 10^4^ mg/L, Ca^2+^/Mg^2+^: 10,872 mg/L) even if complexants are added to stabilize them. However, to obtain polymer gels that are stable under ultra-high-salinity conditions, researchers formed copolymers of acrylamide, AMPS, and NVP, and introduced them into polyacrylamide molecules in large amounts (sometimes up to 90%). This can obviously inhibit the hydrolysis of the amide groups in the molecules, and it also produces good calcium and magnesium resistance.

Among such compounds, the series of commercial SAV polymers produced by the French company Société Nouvelle Floerger (SNF) have been well studied and are considered to have great potential in the field of enhanced oil recovery from ultra-high-temperature and ultra-high-saline oil reservoirs. Two representative polymers, SAV10 and SAV55, were thus obtained from SNF and used to investigate the feasibility of using these types of polymers to form stable gels in ultra-high-saline conditions.

#### 2.3.1. Optimization of the Polymers (SAV10 and SAV55)

Table 5 presents the syneresis rates and elastic moduli measured for the SAV10 and SAV55 polymer gels under harsh conditions (125 °C; TDS: 22.5 × 10^4^ mg/L, Ca^2+^/Mg^2+^: 10,872 mg/L). It can be seen that the two polymer gels showed no signs of syneresis even after aging for 30 days in harsh conditions. Furthermore, the gels were stable even without the assistance of an added complexant.

The AMPS content in SAV10 is high (up to 90%) and the gels formed are relatively weak (elastic moduli of 106.0 and 361.0 mPa). The polymer SAV55 has a lower AMPS content (around 60%) and its gels are subsequently stronger (elastic moduli of 469.0 and 1860 mPa). However, the SAV10 and SAV55 polymer gels are all stable under the conditions used. This means that for use in ultra-high-saline conditions, it is not necessarily true that it is better to have higher AMPS content in the polymer. This is because the higher the AMPS content, the more expensive it is to produce the polymer and the lower the strength of the gel formed. A more appropriate AMPS content might be ~60%. This would help reduce production costs, improve the strength of the polymer gel, and still ensure that the polymer gel remains stable (no syneresis) under HTHS conditions. We also extended this stability evaluation to 90 days, and still no significant syneresis occurred in the aforementioned SAV polymer gels.

#### 2.3.2. Plugging Capacity of Gels

**Figure 6:**
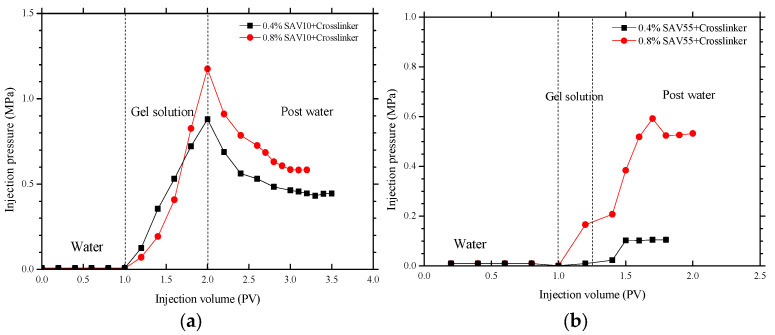
The plugging pressures measured using: (**a**) SAV10 gel in 1000 mD cores, and (**b**) SAV55 gel in 5000 mD cores.

The SAV-based gels are much weaker (Table 5) than the polymer gels based on the conventional polymers HPAM, CPAM, AP-P5, and MKY (Table 2 and Table 4). This is because the SAV10 and SAV55 polymers have small molecules and contain a large number of AMPS groups, which reduce the number of amide groups capable of crosslinking with the phenolic system. We therefore needed to determine whether or not these low-strength gels can effectively plug the pores in oil-bearing media. Thus, plugging experiments were performed using the SAV10 and SAV55 gels and cores with permeabilities of 1000 and 5000 mD. The results obtained are shown in Figure 6.

Figure 6a shows that the injection pressures increased significantly as the SAV10 gel solutions were injected into the 1000 mD cores (starting from the initial values of 0.006 and 0.008 MPa after the previous water injection step). The pressures ultimately rose to 0.880 and 1.175 MPa, respectively, at the end of the gel injection step; the maximum resistance factors are 146.7 and 146.8, respectively. After aging 24 h for gelation, the experiments were resumed and the post-water-injection pressure gradually decreased and then stabilized at 0.445 and 0.583 MPa (corresponding to calculated residual resistance factors of 74.0 and 72.9, respectively). These results clearly indicate that the gels were able to effectively block the high-permeability channels in the cores.

For the higher-permeability cores (5000 mD), we switched to polymer gels based on SAV55 (at polymer concentrations of 0.4% and 0.8%) and performed plugging experiments using a smaller amount of gel (injection volume of 0.3 PV). After the initial water-injection step, the injection pressures were very low (0.001 MPa in both cases). The injection pressures then rose sharply upon treatment with the gel, attaining maximum values of 0.105 and 0.532 MPa, respectively (in the postwater stage). The residual resistance factors were subsequently calculated to be very high: 105.0 and 532.0, respectively.

Our results indicate that SAV10 and SAV55 gels can efficiently block high-permeability channels even though they are weaker in strength compared to traditional HPAM, CPAM, and AP-P4 gels (compare Table 2, Table 4 and Table 5). They therefore have good application potential in HTHS reservoirs. More importantly, we have now obtained gels that are stable and suitable for use in very harsh environments, that is, temperatures of 125 °C and salinity levels that are ultra-high (TDS: 22.5 × 10^4^ mg/L, Ca^2+^/Mg^2+^: 10,872 mg/L).

As these special calcium- and magnesium-resistant SAV polymer gels are stable in harsh conditions, they may also be suitable for use in medium-saline conditions. However, they are still expensive at the moment (generally three times the price of the ‘ordinary’ polymers). Therefore, complexant-stabilized gels are likely to be the cheapest (and therefore best) choice for mildly saline conditions. The special calcium- and magnesium-resistant SAV polymer gels should be mainly considered for use in more demanding conditions, i.e., reservoirs with ultra-high salinity.

## 3. Conclusions

The poor compatibility of carboxylate groups (formed by the hydrolysis of the amide groups in polymers at high temperature) with Ca^2+^/Mg^2+^ ions is the root cause of gel syneresis. Significant syneresis and destruction of polymer gels does not occur if the conditions just involve either a high temperature (e.g., 125 °C) or a high Ca^2+^/Mg^2+^ concentration. Using a high concentration of AP-P5, MKY, and CPAM polymers or adding a complexant to reduce the effect of Ca^2+^/Mg^2+^ ions on the carboxylic acid groups all work in developing stable polymer gels for medium-saline (TDS: 3.4 × 10^4^ mg/L, Ca^2+^/Mg^2+^: 1650 mg/L) environments, but, the latter is the most suitable and cost-effective method. However, when an ultra-high salinity is encountered, e.g., TDS: 22.5 × 10^4^ mg/L (Ca^2+^/Mg^2+^: 10,872 mg/L), those gels improved by the aforementioned methods all become unstable. In this case, the special calcium- and magnesium-resistant polymers SAV10 and SAV55 are needed (although they are more expensive), because their gels are stable, and while they are relatively low in strength, they can still effectively plug the high-permeability channels in cores. In this way, we have obtained the optimal polymer gels suitable for medium- and ultra-high salinities, respectively. Researchers could directly find an applicable and economical gel for a reservoir corresponding to its salinity, thus, unstable or more expensive gels are avoided.

## 4. Materials and Methods

### 4.1. Materials

A total of seven different commercial polymers were obtained from three companies (Table 6). The crosslinker used to prepare the polymer gel is a typical phenolic system and includes hexamethyltetramine (urotropine) and resorcinol. Thiourea was used as a deoxygenation agent. Complexants were also used as stabilizers, including sodium salicylate, glycine, EDTA, sodium oxalate, and diethylenetriamine penta(methylene phosphonic acid) (DTPMP).

Two saline solutions were also prepared with different amounts of added salts and total dissolved salts (TDSs). One corresponded to a medium-saline solution (with a TDS of 33,645 mg/L) and the other an ultra-high-saline solution (with a TDS of 225,068 mg/L); their specific ionic compositions are shown in Table 7. These samples are intended to represent the salinity conditions typically encountered in real reservoirs. The former, for example, emulates reservoirs with medium salinity levels, e.g., the Shengli class III reservoir and some offshore oilfields such as the Weizhou and Bohai oilfields, and so on. On the other hand, the latter represents reservoirs with ultra-high salinity, e.g., the Tazhong 402 and Tahe oilfields in China and some carbonate–salt reservoirs in the Middle East (such as the Ahadib Oilfield), and so on. To further study the effect of salinity/ion type on the stability of the gels, some other water conditions were also employed (see Table 1 for details). Gel solution: polymers with prespecified concentrations were slowly added to those aforementioned salt waters to make polymer solutions firstly. After that, the crosslinkers (urotropine and resorcinol) and stabilizers (sodium salicylate, glycine, EDTA, etc.) with predesigned type and concentration were added in turn; thus, gel solutions of different compositions were prepared. Then, those solutions were maintained at different temperatures (80 °C and 125 °C) to gel and age for stability evaluation. The model cores used in the final gel-plugging experiments are artificial cores with diameters of 2.5 cm and a length of 10.0 cm. Cores with permeabilities of 1000 and 5000 mD were employed.

A rheometer (Anton Paar MCR 302, Anton Paar Co., Ltd., Vienna, Austria) was used to measure the elastic moduli of the polymer gels and thus determine their strength. The gels were stored in ampoules and subjected to high temperatures in order to observe the extent of their syneresis as a function of time. An air bath was used to provide the high-temperature environment required during the gel-aging and seepage experiments. A core seepage device was used to conduct the gel-plugging experiments and thus determine the ability of the gels to plug the high-permeability water channels in the cores.

### 4.2. Methods

#### 4.2.1. Stability Tests

Syneresis and poor thermal stability are the key problems inhibiting the use of gels in HTHS environments. Therefore, we focused on the stability of gels by investigating their syneresis rates and elastic moduli. The specific experimental steps are as follows: (1) The polymer gel solution is first prepared by adding the polymer, crosslinker, and other additives to the saline water in the required ratios. (2) A certain volume of gel solutions (e.g., 20 mL) are transferred to ampoules which are sealed and maintained at 125 °C to age the gel. (3) Samples are removed at specific intervals, then, one of those ampoules is broken for isolated water collection and measurement; thus, the gel syneresis rate can be calculated (the ratio of the volume of effluent water to the initial volume of gel solution 20 mL). (4) Finally, the gel sample is transferred to the rheometer and its elastic modulus determined to quantify its strength (test conditions: a stress of 0.1 Pa is employed and the frequency used is 1 Hz).

#### 4.2.2. Core-Plugging Tests

**Figure 7:**
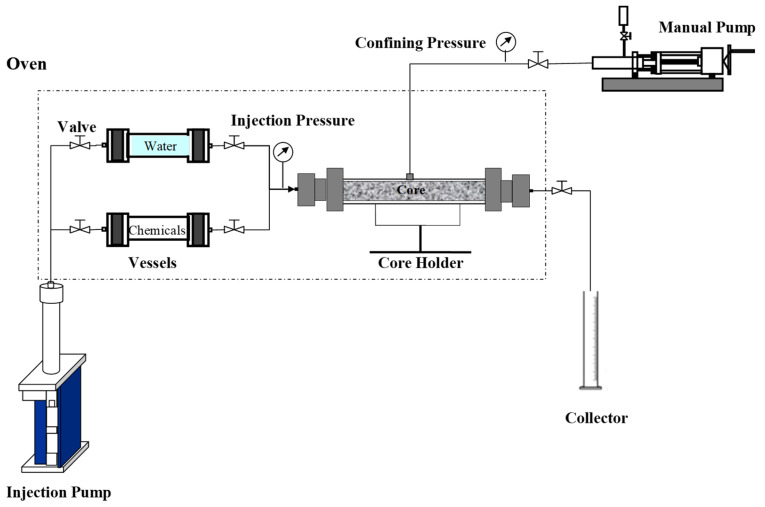
Schematic diagram of the experimental apparatus used in core-plugging experiments.

To assess the ability of the gels to plug high-permeability channels, core-plugging experiments were carried out using prefabricated artificial cores (each of diameter 2.5 cm, length 10.0 cm, and permeability of 1000 or 5000 mD), with the experimental apparatus illustrated in Figure 7.

Two high pressure vessels were employed to store the experimental fluids (water and chemicals–polymer gel solution). An automatic pump was connected to the vessels to inject the fluids into the core holder within which the core was placed (oriented horizontally) to simulate a porous media such as a reservoir. A manual pump was employed to apply a confining pressure to the core holder (maintained at 3.0 MPa above the pressure inside) to avoid the bypassing of the injected fluids from the side of the core. At the end of the process, a collector was used to gather the liquids produced. All experimental pressures were measured using pressure gauges connected to the system. The oven was used to heat the vessels and core holder to the desired experimental temperature (125 °C).

The specific experimental steps employed were as follows: (1) The previously evacuated core was first saturated with water, with its pore volume (PV) calculated from the difference between its wet weight and dry weight. (2) The core was then placed into the core holder, saline water was continuously pumped into the core, and the injection pressure was measured. (3) Gel solution (0.3 or 1.0 PV) was injected into the core, which was then maintained at 125 °C for 12 h to gel and plug it. According to bottle tests, the gelation time of the solution is about 6 h, so the used 12 h here is long enough to ensure its full gelation in the core. (4) Postwater injection was subsequently performed. The injection pressures observed during the entire previous water, gel solution, and post-water-injection processes were recorded to help assess the plugging capacity of the gel.

## Figures and Tables

**Table 1:** Syneresis rates and elastic moduli of gels subjected to different conditions (temperature, ion type, and ion concentration).

No.	Variable Factor	Temperature (°C)	Cation (Concentration in mg/L)	Gel Stability		
				Time (day)	Syneresis Rate (%)	Elastic Modulus (mPa)
1	Temperature	60	Na^+^ (10,686) Ca^2+^/Mg^2+^ (1650)	30	0.0	1986.0
2		80		30	0.0	1360.0
3		100		1	100.0	–
4		125		1	100.0	–
5	Na^+^ conc.	125	Na^+^ (5000)	30	0.0	6100.0
6		125	Na^+^ (20,000)	30	0.0	3860.0
7		125	Na^+^ (40,000)	30	0.0	2350.0
8		125	Na^+^ (80,000)	30	0.0	2120.0
9	Ca^2+^/Mg^2+^ conc.	125	Ca^2+^/Mg^2+^ (200)	30	5.0	894.0
10		125	Ca^2+^/Mg^2+^ (400)	30	5.0	425.0
11		125	Ca^2+^/Mg^2+^ (800)	1	100.0	--
12		125	Ca^2+^/Mg^2+^ (1200)	1	100.0	--
13		125	Ca^2+^/Mg^2+^ (1600)	1	100.0	--

**Table 2:** Syneresis rates and elastic moduli of gels formed by different types of polymers.

No.	Polymer	Gel Composition	Stability		
			Time (day)	Syneresis Rate (%)	Elastic Modulus (mPa)
	HPAM	0.8% HPAM + crosslinker	1	100.0	–
		0.4% HPAM + crosslinker	1	100.0	–
	PAM	0.8% PAM + crosslinker	1	100.0	–
		0.4% PAM + crosslinker	1	100.0	–
	CPAM	0.8% CPAM + crosslinker	30	10.0	8700.0
		0.4% CPAM + crosslinker	1	100.0	–
	AP-P5	0.8% AP-P5 + crosslinker	30	10.0	6410.0
		0.4% AP-P5 + crosslinker	1	100.0	–
	MKY	0.8% MKY + crosslinker	30	10.0	5530.0
		0.4% MKY + crosslinker	2	100.0	–

**Table 3:** Syneresis rates and elastic moduli of gels stabilized using different complexants.

No.	Complexant	Gel Composition	Stability		
			Time (day)	Syneresis Rate (%)	Elastic Modulus (mPa)
1	DTPMP	0.4% PAM + crosslinker + 0.3% DTPMP	30	90.0	–
2	Glycine	0.4% PAM + crosslinker + 0.3% glycine	30	20.0	2986.0
3	EDTA	0.4% PAM + crosslinker + 0.3% EDTA	30	25.0	3206.0
4	Sodium salicylate	0.4% PAM + crosslinker + 0.3% sodium salicylate	30	20.0	3010.0
5	Sodium oxalate	0.4% PAM + crosslinker + 0.3% sodium oxalate	30	10.0	2960.0

**Table 4:** Syneresis rates and elastic moduli of different polymer gels stabilized using sodium oxalate.

No.	Polymer	Gel Composition	Stability		
			Time (day)	Syneresis Rate (%)	Elastic Modulus (mPa)
1	HPAM	0.8% HPAM + crosslinker + 0.4% sodium oxalate	30	0.0	6160.0
2		0.4% HPAM + crosslinker + 0.4% sodium oxalate	30	0.0	1930.0
3	PAM	0.8% PAM + crosslinker +0.4% sodium oxalate	30	0.0	8540.0
4		0.4% PAM + crosslinker + 0.4% sodium oxalate	30	0.0	3230.0
5	CPAM	0.8% CPAM + crosslinker + 0.4% sodium oxalate	30	0.0	8960.0
6		0.4% CPAM + crosslinker + 0.4% sodium oxalate	30	0.0	3620.0
7	AP-P5	0.8% AP-P5 + crosslinker +0.4% sodium oxalate	30	0.0	8720.0
8		0.4% AP-P5 + crosslinker + 0.4% sodium oxalate	30	0.0	3630.0
9	MKY	0.8% MKY + crosslinker + 0.4% sodium oxalate	30	0.0	7460.0
10		0.4% MKY + crosslinker + 0.4% sodium oxalate	30	0.0	2560.0

**Table 5:** Syneresis rates and elastic moduli of gels formed using SAV10 and SAV55 polymers.

No.		Polymer	Gel Composition	Stability		
				Time (day)	Syneresis Rate (%)	Elastic Modulus (mPa)
1	SAV10		0.4% SAV10 + crosslinker	30	0.0	106.0
2			0.8% SAV10 + crosslinker	30	0.0	361.0
3	SAV55		0.4% SAV55 + crosslinker	30	0.0	469.0
4			0.8% SAV55 + crosslinker	30	0.0	1860.0

**Table 6:** The different types of polymers used in this work.

Type	Abbreviation	Molecular Weight (×10^4^)	Hydrolysis Degree (%)	Supplier
Anionic	HPAM	1600	21.3	Hebei Xinxing Chemical Co., Ltd.
Nonionic	PAM	1600	3.1	
Cationic	CPAM	1600	1.4	
Hydrophobic association	AP-P5	1600	-	Sichuan Guangya Polymer Co., Ltd.
	MKY	1600	-	
Anionic	SAV10	300–500	6.0	Ethan Co., France (SNF)
	SAV55		6.0	

**Table 7:** Ionic compositions of the saline solutions used in this work.

No.	Ion Type/Concentration (mg/L)						TDS (mg/L)
	K^+^ + Na^+^	Ca^2+^	Mg^2+^	Cl^−^	SO_4_^2−^	HCO_3_^−^	
1	10,686.0	439.0	1211.0	19,457.0	1619.0	226.0	33,645.0
2	77,974.0	9410.0	1462.0	140,257.0	736.0	337.0	225,068.0

## Data Availability

The data generated and analyzed during this study are available fromthe corresponding author upon reasonable request.
